# A user’s guide to designing efficient and safe mucosal vaccines: Challenges & potentials

**DOI:** 10.1093/oxfimm/iqaf007

**Published:** 2025-08-26

**Authors:** Divya Sinha, Prajwal Kargal Gopalakrishna, Stephane Paul, Stephanie Longet

**Affiliations:** CIRI—Centre International de Recherche en Infectiologie, Team GIMAP, Univ Lyon, Université Claude Bernard Lyon 1, Inserm, U1111, CNRS, UMR5308, CIC 1408 Vaccinology, F42023, Saint-Etienne, France; CIRI—Centre International de Recherche en Infectiologie, Team GIMAP, Univ Lyon, Université Claude Bernard Lyon 1, Inserm, U1111, CNRS, UMR5308, CIC 1408 Vaccinology, F42023, Saint-Etienne, France; CIRI—Centre International de Recherche en Infectiologie, Team GIMAP, Univ Lyon, Université Claude Bernard Lyon 1, Inserm, U1111, CNRS, UMR5308, CIC 1408 Vaccinology, F42023, Saint-Etienne, France; Immunology Department, University Hospital of Saint-Etienne, F42055, Saint-Etienne, France; CIC 1408 Inserm Vaccinology, University Hospital of Saint-Etienne, F42055, Saint-Etienne, France; CIRI—Centre International de Recherche en Infectiologie, Team GIMAP, Univ Lyon, Université Claude Bernard Lyon 1, Inserm, U1111, CNRS, UMR5308, CIC 1408 Vaccinology, F42023, Saint-Etienne, France

**Keywords:** mucosal vaccines, mucosal immunity, vaccine delivery, adjuvants

## Abstract

Mucosal immunization represents a promising approach to protect against pathogens that enter through mucosal surfaces. This review provides a practical overview of the mucosal immune system’s main features and explores the benefits of mucosal vaccination, including its capacity to induce both local and systemic immune responses. Key challenges—such as mechanical barriers, the tolerogenic nature of mucosal immunity and variability due to environmental influences—are examined in detail. Strategies to overcome mucosal tolerance, improve antigen uptake and enhance immunogenicity are discussed, alongside recent advances that combine multiple mucosal routes or explore less conventional pathways. The review also outlines practical considerations for optimizing vaccine delivery and evaluating immune responses, offering a user-oriented guide to the current landscape and future directions in mucosal vaccine development.

## Introduction

World Health Organization’s 2022 report “Imagining the future of pandemics and epidemics” highlighted that several more pandemics are predicted in the future owing to the great impact of increased human population, urbanization and climate change, and would possibly lead to a much greater global mortality and morbidity [1]. The ongoing emergence and evolution of novel pathogens including viruses require promising approaches to strengthen our defense. Mucosal immunity sites are at the forefront of these defenses as mucosal surfaces are the prime targets through which many pathogens invade the human body. As a result, mucosal vaccination presents a paradigm shift in vaccine technology and not only promises enhanced protection but also challenges us to reimagine the future of immunization. Most currently licensed vaccines against mucosal pathogens are administered parenterally and induce protective systemic immune responses but elicit poor antigen-specific immune responses at the mucosa [2–4]. Mucosal vaccination is a promising approach that can elicit strong immune responses in the mucosal tissues lining the respiratory, gastrointestinal, and genitourinary tracts, thereby preventing infection at the entry site while simultaneously generating systemic adaptive immunity [5–7]. Furthermore, mucosal vaccines offer the advantage of being non-invasive, easy to administer, and are ideal for mass vaccinations [8].

### Main features of mucosal tissues

Mucosal surfaces form a system which acts as a mechanical, chemical and biological barrier against environmental insults. This system is composed of unique innate and adaptive immune mechanisms which protects from invasive pathogens. Mucosal surfaces are classified into Type I and Type II mucosa based on specific features. Type I mucosa is found in the intestinal, respiratory and female upper genital tracts, while Type II mucosa is found in cornea, mouth and the female lower genital tract including vagina [9, 10].

One of the main features of mucosal tissues is the presence of epithelium. Within the follicle-associated epithelium, specialized epithelial cells named Microfold (M) cells facilitate antigen uptake and transcytosis, delivering luminal material to underlying immune cells. Below the epithelial layer of mucosal tissue, there is the *lamina propria*. It is a layer of connective tissues which contain numerous cells of innate immune system including macrophages, dendritic cells (DCs), mast cells and innate lymphoid cells able to sense pathogens and activate the adaptive immunity [11]. The *lamina propria* is also composed of Mucosal-Associated Lymphoid Tissues (MALT) (eg Gut-associated Lymphoid Tissues (GALT), Bronchus-associated lymphoid tissues (BALT) and Nasal-associated lymphoid tissues (NALT)) where adaptive immune responses are initiated [12]. The adaptive immune system plays an essential role in maintaining mucosal integrity by orchestrating specific and durable immune responses through the activity of lymphocytes. Antigen-presenting cells (APCs) such as dendritic cells, macrophages and B cells sample and process antigens at mucosal surfaces, presenting them via MHC class I and II molecules to activate CD8^+^ cytotoxic T cells and CD4^+^ helper T cells, respectively [13]. CD4^+^ T cell subsets—including Th1, Th2, Th17, Th22, and regulatory T cells—contribute to pathogen clearance through distinct effector functions. While Th1 and CD8 +responses are primarily directed against intracellular pathogens, Th2, Th17, and Th22 cells mediate immunity to extracellular microbes [11, 13]. In parallel, B cells produce antigen-specific antibodies, particularly secretory IgA (SIgA), which predominates at mucosal sites. SIgA is actively transported across epithelial layers and acts by neutralizing pathogens, preventing microbial adherence and modulating local immune responses [14]. Long-lived memory T and B cells, including tissue-resident T (Trm) and B cells (Brm), may persist at mucosal sites and provide rapid recall responses [15]. Efforts to elicit protective cellular responses, stimulate SIgA production and generate tissue-resident memory responses at mucosal surfaces, should guide the current research towards novel vaccine strategies.

### Benefits of mucosal vaccines

Mucosal vaccination has the potential to generate antigen-specific immunity at the primary sites of infections, preventing further transmission, while contributing to herd immunity [16–18]. Moreover, mucosal vaccination may elicit immune response at the administered site and at other distant mucosal compartments [19]. The interconnected nature of mucosal tissues—commonly referred to as the common mucosal immune system—has important implications for mucosal vaccine design, as immune responses elicited at one mucosal site can influence immunity at distant mucosal surfaces. For instance, in both mice and humans, intranasal vaccination has been reported to generate robust immune responses not only in the upper and lower respiratory tracts but also in distal sites such as the urogenital tract [20, 21]. Oral immunization in humans has been shown to induce immune responses in the salivary glands and tear ducts, with limited but detectable activity in nasal secretions [22]. Intraocular vaccination in mice models have established induction of local immunity in the conjunctiva and tears but can also lead to more distal responses in the saliva, nasal passages, and even the vaginal mucosa [23, 24]. In contrast, intrarectal immunization in mice tends to elicit more localized responses confined to the rectal and intestinal mucosa, making it a suitable strategy for targeting pathogens that infect these specific regions [25–27]. Given this functional compartmentalization of mucosal immune responses based on the route of immunization, it is critical to select the administration route that best aligns with the target site of immune protection. In some cases, combining mucosal routes may be beneficial to broaden or enhance protective coverage.

Furthermore, mucosal vaccination has the potential to promote the development of Trm and Brm cells [28]. These memory subsets are non-circulatory and characterized by long-term tissue residency in peripheral non-lymphoid tissue such as the mucosa [29]. These cells are programmed to respond and elicit rapid recall responses upon antigen encounter and accelerate pathogen clearance [30]. Developing mucosal vaccine strategies to improve the induction of Trm and Brm can be key in the future. Mucosal adjuvanted vaccine strategies may help in efficiently eliciting tissue-resident memory responses [31].

Even though mucosal vaccination could offer immunological and logistics benefits, only a few mucosal vaccines have been approved for human use. Eight oral vaccines exist for poliovirus, rotavirus, *Salmonella typhimurium* and *Vibrio cholerae* while one intranasal vaccine is available against influenza [7, 32]. In addition, India and China approved their first intranasal vaccines, iNCOVACC (BBV154) and Convidecia Air respectively, both recombinant adenovirus-based vaccines [6, 33, 34]. The limited number of licensed mucosal vaccines reflects the significant challenges faced by researchers in their development and evaluation.

## Challenges for an effective mucosal vaccine strategy

### Challenges associated with mechanical barriers

Some of the key characteristics at most mucosal surfaces include the mucosal secretions, such as salivary secretions in the mouth, vaginal and rectal secretions, as well as tear fluids in the ocular cavity. Whilst these features grant us protection, they often present as some of the biggest challenges to mucosal immunization because the secretions can dilute and/or clear out the vaccine components. Another challenge is the presence of epithelial cells with tight junctions that prevent pathogenic invasion [35]. For vaccines, increased antigen uptake is crucial for effective interaction with DCs, leading to their maturation and the subsequent activation of an immune response. However, the success of this process relies on the vaccine antigens being efficiently absorbed by the mucosal surface. Therefore, designing vaccine strategies that enhance epithelial cell uptake or can pass through tight cell junctions is essential for developing effective mucosal vaccine candidates. The strategy selected or designed to cross the epithelial barrier can also be tailored based on the route of vaccination. As previously described, every mucosal tissue has anatomical specificities and may have different types of epithelium further emphasizing that a “one key fit all” approach cannot be the possible solution to address the mucosal barrier and specialization is necessary [36].

The presence of proteolytic enzymes that enrich the mucosal microenvironment is also a challenge. For example, digestive enzymes found in saliva and intestinal tract could potentially degrade the vaccine antigens administered by oral route. Furthermore, the acidic environment at mucosal surfaces, such as the oral cavity, the gastro-intestinal tract and the vaginal mucosa, present in order to prevent the growth and invasion of harmful pathogens and protects the body from infection, can be a big hurdle for successful antigen delivery across mucosal surfaces. The highly acidic environment at most mucosal surfaces can result in the rapid degradation of mucosal vaccine antigens and dampen their effectiveness.

Another key barrier at the mucosal sites for vaccination would be the mucus layer found on most mucosal surfaces. Although helpful in preventing pathogenic invasion, mucus can often be a significant barrier to overcome when it comes to mucosal vaccination. Not only does the mucus layer slow down the diffusion of molecules through it but it also acts as a physical sieve to trap and eliminate small molecules [37]. It can impact absorption, as well as the antigen transport across the mucosal epithelium [38]. Therefore, designing mucosal vaccine strategies that protect the formulation from enzymatic degradation and low pH, while enabling it to cross the epithelial barrier without being rapidly cleared, is crucial for effective mucosal absorption. Potential technologies to address these challenges are discussed in the next sections.

### Challenges associated with mucosal tolerogenic responses

The immune responses to mucosal vaccines are often tricky to induce, since overcoming mucosal tolerogenicity presents as a major challenge. Mucosal tolerogenicity and its extent is site-dependent. For example, the mucosal tolerance at the oral and intestinal mucosa is necessary to prevent an immune response against harmless plethora of antigens consumed via the food we ingest. A predominant set of CD4^+^ T-cells present in the oral mucosa, responsible for this tolerogenic environment, are the regulatory T (Treg) [39]. Treg cells are characterized by their ability to suppress T cell responses by the production of cytokines such as TGFβ, IL-10 and granzyme B, which can induce immune cell death [40]. In addition to Tregs, Th17 cells, producing IL-17 and oral DCs are also involved in oral mucosal tolerance where, in addition to displaying inhibitory surface molecules, the DCs, much like Tregs, also express IL-10 or TGFβ [41]. Interestingly, IL-17 production has been shown to increase with age, and the regulatory mechanisms involving Th17 cells at the oral mucosa appear to differ from those in other mucosal sites, particularly in how they modulate CD4 + T cell effector functions [42]. This distinct immunological environment contributes to the phenomenon of oral tolerance—a key challenge for oral vaccine design. Notably, the antigen dose plays a critical role: high doses of orally delivered antigen may lead to clonal deletion or anergy of antigen-specific T cells, while low doses may still induce anergy rather than activation. In both scenarios, the outcome is a suppressed immune response, highlighting the complexity and the importance of carefully optimizing antigen dose and delivery strategies in oral vaccines [43].

These tolerogenic responses are also found in the nasal mucosa. The human nasal immune tolerant environment is maintained by a balance between Tregs and Th17 cells and surprisingly can also be tolerant to high doses of Lipopolysaccharide exposure. This tolerance could be overcome by stimulation of Fc gamma receptor III and Toll-like Receptor 4 (TLR4) stimulation, as opposed to individual TLR4 stimulation which induced tolerant responses [44, 45]. Another set of key resident immune cells called innate lymphoid cells (ILCs) are also present. Specific subsets of these cells, that is ILC2 in mice and ILC3 in humans are present abundantly in the respiratory mucosa [46]. These cells are ideal for detection of changes within the mucosal microenvironment and respond to either maintain local homeostasis or clear infections [47]. Regulatory ILCs have been identified for the production of TGFβ and IL-10, similar to Th17 cells and to maintain a tolerogenic environment at mucosal surfaces. They have also been implicated to play a role in respiratory autoimmune diseases and allergies [46, 48].

### Mucosal vaccines and the microbiome

In an interesting approach, accounting for local microbiota variables on vaccination responses, a recent study demonstrated that disruption of local nasal bacteria via intranasal antibiotic treatment, prior to intranasal influenza vaccination actually led to enhanced influenza specific IgA responses in the nasal washes and serum IgG levels. Interestingly, depletion of commensal bacteria in the upper respiratory tract significantly lowered viral replication within 2 days after influenza infection [49]. A similar study reported that the intranasal administration of Live Attenuated Influenza Vaccine (LAIV) could alter the nasal microbiome and certain specific alterations can also further influence the production of SIgA at nasal surfaces [50]. This indicates that the nasal microbiota potentially could also play a role in mucosal immunization and needs to be researched at a greater depth. Furthermore, the gut microbiota can also significantly impact the nasal mucosa. Research indicates that the gut microbiota influences the immune health of the nasal mucosa through metabolic byproducts like Short-Chain Fatty Acids (SCFAs). These byproducts can reach the nasal cavity via the bloodstream, affecting its immune barrier. Therefore, an imbalance of the gut microbiota could lead to systemic inflammation, impacting the nasal passages and immune function [51].

It was also demonstrated that the gut microbiome could also impact the immune responses to vaccination. A study in infants receiving a combined oral and inactivated polio vaccine schedule showed marked differences in microbiome composition based on their intestinal secretory IgA responses. Infants who did not develop detectable IgA responses exhibited a microbiota dominated by the phylum *Firmicutes*, while those with robust IgA induction were enriched in *Actinobacteria*, suggesting a potential influence of early microbial colonization on mucosal immune development [52]. A similar trend was observed in the context of oral rotavirus vaccination. Ghanaian infants who failed to mount a protective response (non-responders) had a higher abundance of *Bacteroides* and *Prevotella* species, whereas vaccine responders were enriched in *Streptococcus bovis*-related bacteria—a microbial pattern that positively correlated with IgA titers. Notably, Dutch infants who responded well to the same rotavirus vaccine also showed enrichment in *Streptococcus bovis*-like species, indicating a possible microbiome signature predictive of vaccine responsiveness across populations [53]. In addition, the vaccine efficacy of oral rotaviral vaccines was reported to be much higher for infants in high income countries when compared to infants in low income countries. One possible reason for this discrepancy in vaccine efficacy could be due to a higher burden of enteric disease in low income countries, which could impact the gut microbiome and influence vaccine responses [54–57].

## New clinical and pre-clinical studies addressing barriers to mucosal vaccination

The pursuit of effective mucosal vaccination represents a promising frontier in immunology, offering the potential for more accessible and patient-friendly vaccine delivery methods. However, this innovative approach faces significant challenges that must be addressed through rigorous preclinical and clinical studies. These obstacles include understanding the mechanisms of mucosal immunity, optimizing vaccine formulations and delivery systems, and ensuring safety and efficacy across diverse populations. New research is essential to overcome these hurdles, providing insights that could aid the prevention of infectious diseases and improve global health outcomes. The following section will reflect on current research in preclinical and clinical stages of vaccine development that addresses the barriers to mucosal vaccines discussed above.

### Developing mucosal vaccine delivery strategies

A large range of technologies and vectors have been or are being developed to deliver mucosal vaccine formulations. In this section, you can find some key examples. To address vaccine delivery issues via the oral route, a key barrier is the highly acidic conditions in the stomach which can cause degradation of the vaccine antigen. Encapsulation of vaccine antigen is commonly used to protect it. For example, a polysaccharide-coated nanoparticle-based oral vaccine was developed, using ovalbumin as model antigen. When administered intranasally, this polysaccharide coat, prevented antigen release in the acidic environment and promote a sustained release in the intestine. This resulted in enhanced antigen uptake by the intestinal epithelial cells and macrophages and resulted in high mucosal IgA levels [58]. In a similar approach, use of microcontainers, which are novel polymer-based structures, that can be used as delivery systems for vaccines. These microcontainers are designed with special lids that dissolve when they detect specific pH levels in the body. When the pH changes (e.g. in certain areas of the body), the lids of the microcontainers dissolve, allowing the vaccine inside to be released at the right time and place [59]. These microcontainers were utilized for oral vaccine delivery of *Chlamydia trachomatis* vaccine candidate, which can be degraded due to the acidic pH environment in the gastrointestinal tract (GIT). This vaccine candidate was tested in combination with several mucosal vaccine adjuvants in microcontainer formulation. The formulation adjuvanted with α-GalCer showed higher levels of IFN-γ and IL-17A compared to control groups upon oral prime and booster doses. However, due to the weak nature of the immune response generated, further research into such novel techniques are warranted [60]. Designing specific vaccine carriers, such as modified nanoparticles or novel formulations such as Single Multiple Pills (SMPs), with a polymeric coating, resistant to the harsh acidic environment in the GIT can also be utilized for oral vaccination and further aid in overcoming the gastric acidic barrier hindering immune responses to orally delivered vaccines [61, 62].

Recent studies have also shifted their focus on specialized vaccine delivery platforms to overcome the epithelial cell junction based mucosal barrier by employing the use of microneedle fibers. These fibers, which were composed of electro-spun nanofibers with a polymeric backfill matrix, were designed to load vaccine antigens and release these antigens upon administration on the mucosal cavities such as the buccal cavity. Here, ovalbumin was used as an antigen, co-adjuvanted with CpG-ODN and GM-CSF in mouse models. The vaccines were administered sublingually and buccally using the microneedle fiber technology and compared with oral administration of the same vaccine with swabs as controls. The buccal administration of microneedles fiber design led to higher OVA-specific serum IgG responses. Both routes of administration resulted in high levels of IFN-γ secreting lymphocytes in the spleen. However, the buccal vaccination achieved higher levels of both humoral and cellular responses [63]. In the future, similar studies using such novel delivery systems, but with focus on mucosal immune response markers such as secretory IgA, could also be beneficial.

Other mucosal delivery sites also face similar delivery issues and novel studies are actively progressing towards innovative solutions. One such study focused on the development of mucoadhesive chitosan-based vaccine adjuvants expressing inactive Porcine Epidemic Diarrhea Virus, which due to their enhanced retention time in the nasal mucosa were able to actively recruit DCs and macrophages, further improving the antigen permeability after intranasal immunization in mouse model [64]. Polymeric molecules such as Polyethyleneimine, Dimethyl-β-cyclodextrin, and Chitosan were evaluated for their ability to enhance the penetration of drugs across mucosal surfaces. These polymers were tested as drug delivery systems for mucosal administration through intranasal, intravaginal and sublingual routes in mouse models. The study also examined their effectiveness when combined with antigens like recombinant HIV-1 envelope glycoprotein and tetanus toxoid in mice. Notably, when these epithelial penetration enhancers were used in combination with the antigens, they significantly increased antigen-specific IgG and IgA antibody levels in both serum and vaginal samples, compared to using the antigens alone in intranasal and sublingual vaccinations [65].

Penetrating the thick layer of mucus present on mucosal surfaces is key for effective antigen uptake. Nanoparticles have appeared at the forefront, utilizing their small size and ability to act as a vaccine carrier or delivery system. These nanoparticles can be coated with mucus penetrating materials such as water-soluble polymers like peptidoglycan and show enhanced penetration in human cervicovaginal mucus. Interestingly, studies reported the importance of size control for these nanoparticles. 200–500 nm range particles showed effective diffusion but 100 nm particles showed a lower diffusion rate in the cervicovaginal mucus [65]. Furthermore, nanoparticle-based vaccine candidates, when encapsulated by molecules such as Poly-Lactic Acid, demonstrated enhanced mucus penetration in the mouse intestinal mucosa and epithelial barrier crossing via M-cells [66]. Nanoparticles have also been tested for the development of nanocarriers suitable for intravaginal use. Notably, polysuccinimide-based nanocarriers exhibit both acid resistance and the ability to penetrate mucus making them effective for transporting siRNA against sexually transmitted viruses like HIV and HSV-2. By safeguarding siRNA from degradation and facilitating its uptake by vaginal tissues, these nanocarriers offer a promising approach to prevent viral infections at their entry point [67]. This approach can also be incorporated in DNA- and mRNA-based vaccines where peptide-poloxamine-based nanoparticles can be used as carriers for plasmid DNA for transport across the mucus layer in the respiratory mucosa [68]. In another innovative approach, use of non-viral charge reversal nanoparticles that can mimic a virus infecting broad cell types, was developed to express human papillomavirus type 16 L1 gene and was successful in inducing cellular and humoral immunity upon intravaginal administration [69].

Associated with the development of delivery systems, the induction of immune responses can be improved by targeting specific mucosal cells. For example, targeting M-cells for enhancing the specific delivery in intestinal or respiratory tissues might be essential for specific immune responses produced post-mucosal vaccination. One such study focused on the production of M-cell targeting orally delivered vaccines using Lactic acid bacteria (LAB) as carrier molecule expressing the model antigen BmpB via cytoplasmic expression, carrying specific M-cell targeting moiety in mouse models. M-cell targeting enhanced serum IgG and fecal IgA to a significantly higher level as compared to the vaccine formulation devoid of the M-cell targeting moiety. Furthermore, a marked increase in IgA production at the lamina propria was also noted, indicating the advantages of targeting M-cells in the gut to induce significant immune responses [70]. Another strategy involves adapting mechanisms used by certain microbes to invade the GIT, such as exploiting specific intestinal lymphatic pathways. *Saccharomyces* components can be leveraged for their ability to target M-cells. In this approach, glucan cores from *Saccharomyces species* were used to create nanocores, which were then incorporated into mesoporous silica nanoparticles. These nanoparticles, carrying ovalbumin as a model antigen and a tyrosine kinase inhibitor, were able to cross M-cells via Dectin-1 receptors when administered orally and effectively targeted specific sites in C57BL/6 mice [71]. Table 1 refers to the summary of the studies mentioned in this section.

**Table 1.** Summarizing selected technologies and delivery platforms used in mucosal vaccine development, including delivery method, formulation, route, and observed immune responses

Intended goal	Strategy/Platform	Description	Antigen/model used	Route	Immune outcome/key findings	References
Acid protection/pH-responsive delivery	Polysaccharide-coated nanoparticles	Protects antigen from acid; enables sustained release in intestine	Ovalbumin	Oral (via nasal in model)	Enhanced antigen uptake by IECs and macrophages; increased mucosal IgA	[58]
	Microcontainers with pH-sensitive lids	Release antigens at site-specific pH in GIT	*C. trachomatis* + α-GalCer, others	Oral	↑ IFN-γ, IL-17A; weak response; needs improvement	[59, 60]
	Polymeric carriers/“Single Multiple Pills”	Acid-resistant formulations using polymer coatings	Various	Oral	Protected from gastric degradation; targeted delivery	[61, 62]
Mucosal barrier penetration	Mucosal penetration enhancers	Enhance permeability across mucosal surfaces	HIV Env, Tetanus toxoid	Intranasal, Sublingual, Vaginal	↑ serum and mucosal IgA/IgG versus antigen alone	[65]
	Mucus-penetrating nanoparticles	Use peptidoglycan/PLA to cross mucus layers	Model antigens	Vaginal, Intestinal	200–500 nm size optimal for penetration and M-cell transport	
	Polysuccinimide-based nanocarriers	Acid/mucus-resistant siRNA delivery system	siRNA (HIV/HSV-2)	Intravaginal	Enhanced tissue uptake; viral prevention	[67]
	Poloxamine-based DNA delivery	Nanocarriers for gene vaccines crossing mucus	Plasmid DNA	Respiratory	Effective mRNA/DNA transport across mucosa	[68]
M-cell targeting	LAB-expressed M-cell ligand	LAB expressing BmpB + M-cell ligand enhances gut targeting	BmpB	Oral	↑ serum IgG, fecal and lamina propria IgA	[70]
	Saccharomyces-derived nanocores	Dectin-1-targeting silica nanoparticles	Ovalbumin + kinase inhibitor	Oral	Enhanced M-cell uptake; lymphatic targeting	[71]
Mucosal-site specific delivery	Microneedle fibers	In situ nanofiber antigen delivery	Ovalbumin + CpG-ODN + GM-CSF	Buccal/Sublingual	↑ serum IgG, splenic IFN-γ; buccal > swab	[63]
	Chitosan-based mucoadhesives	Enhances nasal retention & APC recruitment	Inactivated PEDV	Intranasal	Improved nasal permeability & APC activation	[64]
Advanced targeting/modulation	Charge-reversal nanoparticles	Mimic viruses for broad cell targeting	HPV16 L1 gene	Intravaginal	Strong cellular and humoral responses	[69]

### Overcoming mucosal tolerance

Overcoming mucosal tolerance remains a major challenge and the use of adjuvants can be a solution. However, one of the major barriers is the lack of mucosal adjuvants approved for human use. Cholera Toxin is the gold standard of mucosal adjuvant to overcome tolerogenicity and boost mucosal immune responses, and has been quite successful in preclinical models [72, 73]. However, due to the toxicity issues surrounding this adjuvant, it might not be the best adjuvant to supplement in a vaccine candidate for human use, particularly via the mucosal route [74]. Recent research has been actively working to address this dearth in mucosal adjuvants. A large range of micro and nanoparticles, modified toxins, liposomes, glycolipids, cytokines have been tested in preclinical models [32]. However, only a few reaches clinical trial evaluation. Use of bacterial-like particles, such as those derived from food-grade non-pathogenic strains of *Lactobacillus lactis* were incorporated in an intranasal flu vaccine as a safe and efficacious mucosal vaccine adjuvant, tested in a phase I human clinical trial resulting in seroconversion of more than 40% trial subjects and increased nasal IgA levels following vaccination [75]. In a phase II trial, the safety and immunogenicity of an intranasal influenza vaccine using heat labile toxin (LT)-derived from *E. coli* (LTh(αK)), as an adjuvant were evaluated. The LTh(αK)-adjuvanted formulation induced significantly higher mucosal IgA antibody production compared to the control and showed long-term mucosal immune responses as mucosal anti-influenza virus hemagglutinin (HA) IgA titers, which remained at least up to 40% at the end of 180 days, indicating potential long-term protection [76]. Another human clinical trial explored the safety and immunogenicity of an intranasal influenza vaccine adjuvanted with gram-positive enhancer matrix derived from *Lactococcus lactis,* produced by heat-acid treatment of the bacteria to remove DNA and other bacterial proteins [77]. The trial reported a mean increase in the serum hemagglutination inhibition (HI) antibody titers and mucosal IgA, although immune responses were lower in older adults [75]. Similarly, a clinical trial assessed ETVAX^®^, an oral vaccine for enterotoxigenic *E. coli*, combined with a novel adjuvant: double mutant heat-labile enterotoxin. The vaccine induced high antigen-specific plasma IgA and IgG levels in both adults and children. Interestingly, the adjuvanted vaccine led to stronger IgA responses in children, particularly with the 1/4 dose, compared to lower doses or placebo. However, data on SIgA levels, which are critical for assessing mucosal immunity, were insufficient [78]. Recently, bacterial derived flagellin has also appeared at the forefront of mucosal vaccine research. Flagellin, a locomotory protein for gram negative bacteria has been actively tested for its ability to activate TLR receptors in the host and the induction of an immune response, post mucosal vaccination [79]. Flagellin, derived from *E. coli species*, was used as an adjuvant in combination with the RSV phosphoprotein antigen. This formulation, delivered intranasally in mice, significantly reduced RSV replication and viral load post-challenge, while avoiding the development of vaccine-enhanced disease [80]. A similar success with flagellin was observed in the development of a therapeutic mucosal vaccine candidate for Alzheimer’s disease. In this study, flagellin derived from *V. vulnificus* was combined with tau protein antigens and administered intranasally to mice. The treatment led to a significant reduction in pathological tau protein accumulation in the brain, improved working memory, and increased survival rates. However, SIgA levels were not reported [81].

Currently, a lot of efforts is placed into the screening and development of mucosal adjuvants at the preclinical level. Furthermore, adjuvant research is also pertinent to address another significant challenge to mucosal vaccination, the lack of approved subunit-based mucosal vaccines. As mentioned previously, most mucosal vaccine candidates are based on live attenuated vaccines. This challenge has also been actively addressed in several studies such as utilizing polyelectrolyte complex-based nanoparticles as adjuvants with H1N1 influenza subunit vaccine, as an intranasal vaccine candidate, resulting in significant induction of mucosal immune responses [82]. Pre-clinical studies direct fusion of *E. coli* enterotoxin A subunit with SARS-CoV-2 receptor-binding domain (RBD) when administered intranasally in mice, also resulted in potent neutralizing antibodies against several strains of SARS-CoV-2 and high IgA titers in bronchoalveolar lavages [83]. Furthermore, in an attempt to develop a broadly acting subunit based influenza vaccine, Stimulator of Interferon Genes or STING agonist were also utilized for their ability to induce potent mucosal immune responses [84, 85]. Varma et.al developed a gel based intranasal influenza vaccine candidate using a novel broadly acting influenza antigen in combination with cGAMP, a STING agonist, which resulted in high serum IgG and SIgA levels [86]. In search of novel molecules, researches have explored, type III interferons, particularly IFN-λ for its potential adjuvant properties in the context of mucosal vaccinations. Due to the presence of IFN-λ receptors on epithelial cells, the anti-viral functions of IFN-λ are mostly dominant at mucosal surfaces [87]. In mice lacking IFN-λ signaling, both CD8^+^ T cell responses and antibody production were weakened after infection with a live-attenuated influenza virus. When Influenza A M2 protein antigen was combined with IFN-λ2 molecule and administered intranasally to mice, this IFN-λ triggered thymic stromal lymphopoietin (TSLP) production in the upper airways, which activated dendritic cells and enhanced the serum IgG levels and the IgA levels in the BAL. This IFN-λ-TSLP pathway specifically improved mucosal immune responses following intranasal vaccination and were not observed for subcutaneous or intraperitoneal vaccination [88]. Recently, its role has also been implicated in the generation of SARS-CoV-2 specific CD8 T-cells in mice which further stresses on the potential of IFN-λ as a novel and effective mucosal vaccine adjuvant [89].

Interestingly, in an attempt to deal with cold-chain maintenance issues, as well as the low immunogenicity of subunit vaccines, researchers also assessed the efficacy of an inhaled intranasal subunit SARS-CoV-2 vaccine, combined with poly I: C and all-trans retinoic acid as adjuvants. This resulted in 100% protection in intranasal/intratracheal administration in mice, even at low doses. This vaccine candidate is now being subjected to further processing to obtain a powder-based formulation to overcome cold-chain maintenance issues occurring during vaccine transport and will further be tested in clinical trials [90].

### Combining several routes of mucosal immunization

An important barrier to mucosal vaccination that needs to be addressed is the unequal immune response at different mucosal sites following mucosal immunization. The combination of different routes of administration could help to induce strong protective responses at specific mucosal sites. Various combinations have been tested in several preclinical models. For instance, administering a recombinant BCG vaccine expressing the V3 sequences of the Japanese consensus HIV (rBCG–pSOV3J1) or HIV-V3J1 antigen through both intrarectal and intradermal routes led to high systemic titers of HIV-1-specific IgG and IgA. Interestingly, this dual-route immunization also triggered delayed-type hypersensitivity reactions against both the HIV-V3J1 peptide and tuberculin in immunized guinea pigs, illustrates the potential benefits of heterologous vaccine administration [91]. A similar study combined the potential of two mucosal immunization routes by comparing the immune response generated using a combination of intranasal and intravaginal mucosal administration. Mice were primed with plasmid DNA encoding HIV virus-like particles (pVLPs) via the intranasal route, followed by intravaginal boosts with HIV-VLPs. This mucosal approach was compared to a parenteral strategy involving intradermal electroporation and subcutaneous administration. While the parenteral route generated stronger systemic T-cell responses, the combined intranasal and intravaginal mucosal administration induced significantly higher T-cell responses in the vaginal mucosa and draining lymph nodes as well as a significant IgA response in both serum and vaginal washes, with neutralizing activity in the latter samples. These findings emphasize the effectiveness of combining intranasal and intravaginal routes in eliciting robust HIV-specific T-cell responses in the vaginal mucosa [92].

Sublingual vaccination is another interesting immunization route as it can bypass the GIT and induces immunity in both the respiratory and genital tracts with limited adverse effects [93]. Intranasal and sublingual immunization of mice with recombinant Group A Streptococcus antigen induced effective systemic IgG and mucosal IgA responses, while subcutaneous immunization generated only serum IgG response [94].

**Figure 1.**
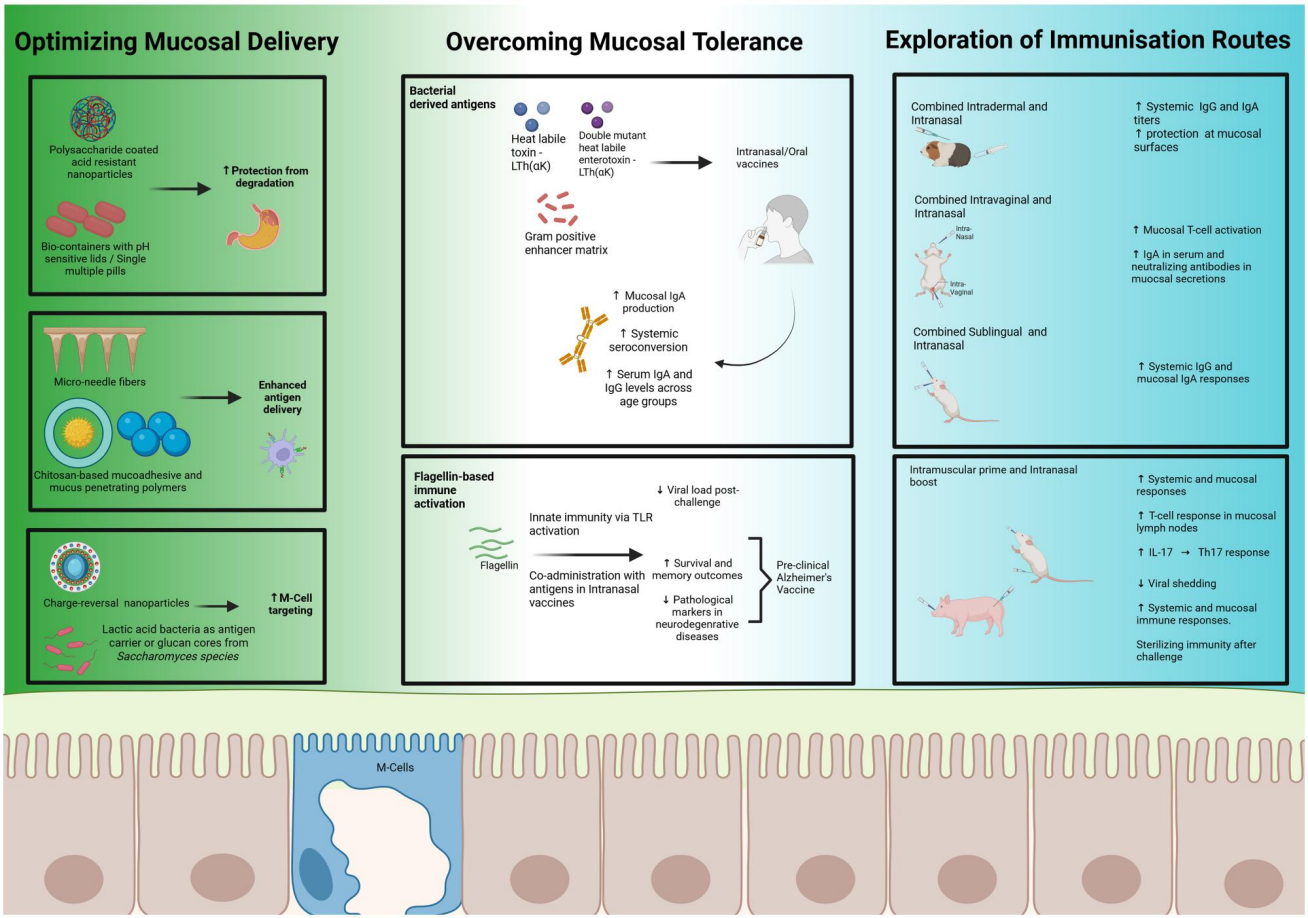
A summary of strategies to enhance mucosal immune responses. This figure summarizes key strategies to enhance mucosal vaccine efficacy. It encompasses innovations in mucosal delivery systems, approaches to overcome mucosal tolerance using microbial vectors, and the exploration of combined mucosal and systemic immunization routes to elicit robust and broad immune responses. Created with BioRender.com.

In recent years, several “Prime and Pull” strategies have been employed to leverage the complementary strengths of intramuscular (IM) and intranasal (IN) vaccines within unified immunization regimens. A notable example was the study by Laghlali *et al*., which evaluated a heterologous prime-boost approach combining an intramuscular priming dose of the SARS-CoV-2 BNT162b2 mRNA vaccine with an intranasal booster comprising the S protein formulated with a novel adjuvant platform, NE/IVT. This platform included a nano-emulsion (NE) and an RNA-based RIG-I agonist (IVT), designed to stimulate robust mucosal immunity. Compared to homologous IM/IM and IN/IN regimens in murine models, the heterologous strategy elicited superior mucosal and systemic antibody responses and significantly enhanced antigen-specific T cell responses in mucosal-draining lymph nodes. Notably, it was the only regimen that conferred sterilizing immunity in the upper respiratory tract, driven in part by elevated IL-17A levels and a skewing towards Th17 responses, which are essential for mucosal defense [95]. A similar principle has been explored in the development of influenza vaccines. In a study conducted in pigs, researchers investigated a prime-boost regimen combining an intramuscular priming dose using a hemagglutinin-encoding propagation-defective vesicular stomatitis viral vector with an intranasal boost using a modified LAIV of the 2009 pandemic H1N1 virus with a truncated NS1 protein. While the LAIV alone resulted in prolonged viral shedding, the heterologous prime-boost approach not only curtailed shedding but also enhanced both systemic and mucosal immune responses. This dual-route strategy successfully induced sterilizing immunity upon challenge with the wild-type influenza virus [96]. These findings showcase the growing potential of combining several routes of vaccines, leveraging mucosal immunizations to enhance protective immunity at both systemic and mucosal levels—offering a promising avenue for the development of next-generation vaccines. Figure 1 provides a comprehensive overview of mucosal vaccine optimization strategies, highlighting advances in delivery technologies, immune modulation, and administration routes as discussed above.

### Unexplored routes of mucosal immunization

Alternative, less explored routes of mucosal vaccination may represent viable options, given the promising results observed in preclinical models. However, in developing mucosal vaccines for human use, it is essential to balance immunological benefits with population acceptability.

Ocular conjunctiva is another mucosal site which is protected via the maintenance of an immune tolerogenic environment. Inflammatory responses in the ocular mucosa can lead to photoreceptor damage and can lead to pathologies involving CD4^+^ T cells and macrophages [97]. The ocular mucosa has several different mechanisms to prevent damage associated with inflammation and antigen exposure which has been previously described in detail [98]. Interestingly, reports of ocular inflammation and uveitis have been previously reported with SARS-CoV-2 vaccination but was relatively uncommon [34, 99]. Although ocular route of mucosal vaccination might not be as popular in humans trials and pre-clinical studies, this route of immunization has been quite successful in inducing protective immune responses in chickens following intra-ocular avian influenza vaccination, thus it could be used as a potential vaccination strategy to prevent avian influenza and can curbs potential spillovers in human populations [100, 101]. In another study, chickens vaccinated with liposomal inactivated avian pathogenic *E. coli* by eye drop produced anti-lipopolysaccharide IgG antibodies in their sera as well as IgA antibodies in their oral mucosa [102]. Vaccination with the commercially available human papillomavirus (HPV) vaccine, Cervarix, as eyedrops in mice induced a robust immune response with anti-HPV antigen-specific IgG and IgA antibodies. These results demonstrates the vaccine’s potential as an effective intra-ocular mucosal vaccine against HPV infections [23].

The rectal route of immunization has also shown promising results against enteric pathogens by inducing appropriate immune effectors in the intestinal mucosa [102–105] Intrarectal immunization with recombinant vaccinia virus expressing carcinoembryonic antigen (CEA) has shown promising results in preventing the progression towards invasive colorectal cancer and inducing CEA-specific local mucosal and systemic humoral and cell-mediated immunity [106]. Rectal administration of Leishmania cells was shown to induce protective Th1-associated IgG2a-biased responses in mice with a potential to be used as a vaccine platform against viral diseases [107].

## Conclusion

In conclusion, the future of mucosal vaccination holds significant potential for revolutionizing global immunization strategies, particularly in the context of emerging infectious diseases. This review aimed to elucidate the key barriers to the effective development of mucosal vaccination and explored potential strategies to overcome these challenges (summarized in Fig. 1). The current literature highlights several critical obstacles, including issues with vaccine delivery due to the inherent nature of the mucosa, tolerogenic and variable immune responses at mucosal sites.

The development of novel mucosal delivery systems and adjuvants represent a significant leap forward in overcoming some of the mucosal barriers discussed in this review. Additionally, the exploration of alternative mucosal routes, such as ocular and rectal immunization, could be an effective means of generating durable mucosal protection to specific pathogens that utilize these mucosal surfaces for invasion.

Looking ahead, integrating mucosal vaccines into global immunization programs could reform public health, particularly in low-income settings where traditional vaccine delivery methods face significant challenges. As global vaccine research and innovation continue to advance, mucosal vaccines are set to play a vital role in future public health immunization strategies, proving to be crucial in the fight against infectious diseases.

## Data Availability

No new data were generated or analysed in support of this research.

## References

[1] World Health Organization. Imagining the future of pandemics and epidemics: a 2022 perspective. Geneva: WHO, 2022, 65. https://www.who.int/publications/i/item/9789240052093

[2] Mao T , Israelow B, Peña-Hernández MA. et al Unadjuvanted intranasal spike vaccine elicits protective mucosal immunity against sarbecoviruses. Science 2022;378:eabo2523. 36302057 10.1126/science.abo2523 PMC9798903

[3] Pilapitiya D , Wheatley AK, Tan H-X. Mucosal vaccines for SARS-CoV-2: triumph of hope over experience. eBioMedicine 2023;92:104585. 10.1016/j.ebiom.2023.104585 37146404 PMC10154910

[4] Prior JT , Limbert VM, Horowitz RM. et al Establishment of isotype-switched, antigen-specific B cells in multiple mucosal tissues using non-mucosal immunization. NPJ Vaccines 2023;8:80. 37258506 10.1038/s41541-023-00677-z PMC10231862

[5] Fragoso-Saavedra M , Vega-López MA. Induction of mucosal immunity against pathogens by using recombinant baculoviral vectors: mechanisms, advantages, and limitations. J Leukoc Biol 2020;108:835–50. 32392638 10.1002/JLB.4MR0320-488R

[6] Huang M , Zhang M, Zhu H. et al Mucosal vaccine delivery: A focus on the breakthrough of specific barriers. Acta Pharm Sin B 2022;12:3456–74. 35818435 10.1016/j.apsb.2022.07.002 PMC9259023

[7] Song Y , Mehl F, Zeichner SL. Vaccine strategies to elicit mucosal immunity. Vaccines (Basel) 2024;12:191. 38400174 10.3390/vaccines12020191 PMC10892965

[8] Luria-Pérez R , Sánchez-Vargas LA, Muñoz-López P. et al Mucosal vaccination: a promising alternative against flaviviruses. Front Cell Infect Microbiol 2022;12:887729. 35782117 10.3389/fcimb.2022.887729 PMC9241634

[9] Kim S-H , Lee K-Y, Jang Y-S. Mucosal immune system and M Cell-targeting Strategies for Oral Mucosal Vaccination. Immune Netw 2012;12:165–75. 23213309 10.4110/in.2012.12.5.165 PMC3509160

[10] Wang Y , Sui Y, Kato S. et al Vaginal type-II mucosa is an inductive site for primary CD8+ T-cell mucosal immunity. Nat Commun 2015;6:6100. 25600442 10.1038/ncomms7100 PMC4348041

[11] Leceta J , Del Campo R, Jordan S. et al Editorial: Immunoregulation at mucosal surfaces. Front Immunol 2022;13:983201. 36032144 10.3389/fimmu.2022.983201 PMC9400381

[12] Elmore SA. Enhanced histopathology of mucosa-associated lymphoid tissue. Toxicol Pathol 2006;34:687–96. 17067953 10.1080/01926230600939989 PMC1797898

[13] Wang R , Lan C, Benlagha K. et al The interaction of innate immune and adaptive immune system. Medcomm (2020) 2024;5:e714. 39286776 10.1002/mco2.714 PMC11401974

[14] Sinha D , Yaugel-Novoa M, Waeckel L. et al Unmasking the potential of secretory IgA and its pivotal role in protection from respiratory viruses. Antiviral Res 2024;223:105823. 38331200 10.1016/j.antiviral.2024.105823

[15] Mettelman RC , Allen EK, Thomas PG. Mucosal immune responses to infection and vaccination in the respiratory tract. Immunity 2022;55:749–80. 35545027 10.1016/j.immuni.2022.04.013 PMC9087965

[16] Tang J , Zeng C, Cox TM. et al Respiratory mucosal immunity against SARS-CoV-2 after mRNA vaccination. Sci Immunol 2022;7:eadd4853. 35857583 10.1126/sciimmunol.add4853 PMC9348751

[17] Focosi D , Maggi F, Casadevall A. Mucosal vaccines, sterilizing immunity, and the future of SARS-CoV-2 virulence. Viruses 2022;14:187. 35215783 10.3390/v14020187 PMC8878800

[18] Flemming A. “Prime and spike” induces mucosal immunity and reduces SARS-CoV-2 transmission. Nat Rev Immunol 2022;22:718. 10.1038/s41577-022-00804-2 PMC9643939 36348057

[19] Nizard M , Diniz MO, Roussel H. et al Mucosal vaccines: novel strategies and applications for the control of pathogens and tumors at mucosal sites. Hum Vaccin Immunother 2014;10:2175–87. 25424921 10.4161/hv.29269 PMC4896761

[20] Rudin A , Riise GC, Holmgren J. Antibody responses in the lower respiratory tract and male urogenital tract in humans after nasal and oral vaccination with cholera toxin B subunit. Infect Immun 1999;67:2884–90. 10338495 10.1128/iai.67.6.2884-2890.1999 PMC96596

[21] Liu Y , Hammer LA, Daamen J. et al Microencapsulated IL-12 drives genital tract immune responses to intranasal gonococcal outer membrane vesicle vaccine and induces resistance to vaginal infection with diverse strains of neisseria gonorrhoeae. mSphere 2023;8:e0038822. 36537786 10.1128/msphere.00388-22 PMC9942569

[22] Bergmann KC , Waldman RH, Tischner H. et al Antibody in tears, saliva and nasal secretions following oral immunization of humans with inactivated influenza virus vaccine. Int Arch Allergy Appl Immunol 1986;80:107–9. 3957442 10.1159/000234034

[23] Kim J , Kim E-D, Shin H-S. et al Effectiveness and safety of injectable human papilloma virus vaccine administered as eyedrops. Vaccine 2023;41:92–100. 36402660 10.1016/j.vaccine.2022.09.070

[24] Seo KY , Han S, Cha H-R. et al Eye mucosa: an efficient vaccine delivery route for inducing protective immunity. J Immunol Baltim Immunol 2010;185:3610–9. 10.4049/jimmunol.1000680 20709955

[25] Agnello D , Hervé CA, Lavaux A. et al Intrarectal immunization with rotavirus 2/6 virus-like particles induces an antirotavirus immune response localized in the intestinal mucosa and protects against rotavirus infection in mice. J Virol 2006;80:3823–32. 16571799 10.1128/JVI.80.8.3823-3832.2006 PMC1440434

[26] He Q , Jiang L, Cao K. et al A systemic prime–intrarectal pull strategy raises rectum-resident CD8+ T cells for effective protection in a murine model of LM-OVA infection. Front Immunol 2020;11:571248. 10.3389/fimmu.2020.571248 33072113 PMC7541937

[27] Agnello D , Denimal D, Lavaux A. et al Intrarectal immunization and IgA antibody-secreting cell homing to the small intestine. J Immunol 2013;190:4836–47. 23547118 10.4049/jimmunol.1202979

[28] Zens KD , Chen JK, Farber DL. Vaccine-generated lung tissue-resident memory T cells provide heterosubtypic protection to influenza infection. JCI Insight 2016;1:e8583285832. 10.1172/jci.insight.85832 PMC4959801 27468427

[29] Lange J , Rivera-Ballesteros O, Buggert M. Human mucosal tissue-resident memory T cells in health and disease. Mucosal Immunol 2022;15:389–97. 34743182 10.1038/s41385-021-00467-7 PMC8571012

[30] Rotrosen E , Kupper TS. Assessing the generation of tissue resident memory T cells by vaccines. Nat Rev Immunol 2023;23:655–65. 37002288 10.1038/s41577-023-00853-1 PMC10064963

[31] Longet S , Paul S. Pivotal role of tissue-resident memory lymphocytes in the control of mucosal infections: can mucosal vaccination induce protective tissue-resident memory T and B cells? Front Immunol 2023;14:1216402. 10.3389/fimmu.2023.1216402 37753095 PMC10518612

[32] Lavelle EC , Ward RW. Mucosal vaccines–fortifying the frontiers. Nat Rev Immunol 2022;22:236–50. 34312520 10.1038/s41577-021-00583-2 PMC8312369

[33] Halperin SA , Ye L, MacKinnon-Cameron D. et al; CanSino COVID-19 Global Efficacy Study Group. Final efficacy analysis, interim safety analysis, and immunogenicity of a single dose of recombinant novel coronavirus vaccine (adenovirus type 5 vector) in adults 18 years and older: an international, multicentre, randomised, double-blinded, placebo-controlled phase 3 trial. Lancet Lond Lancet 2022;399:237–48. 10.1016/S0140-6736(21)02753-7 PMC8700283 34953526

[34] Singh RB , Parmar UPS, Kahale F. et al Vaccine-associated uveitis after COVID-19 vaccination. Ophthalmology 2023;130:179–86. 36055601 10.1016/j.ophtha.2022.08.027 PMC9428109

[35] Tugizov S. Virus-associated disruption of mucosal epithelial tight junctions and its role in viral transmission and spread. Tissue Barriers 2021;9:1943274. 34241579 10.1080/21688370.2021.1943274 PMC8794520

[36] Pelaez-Prestel HF , Sanchez-Trincado JL, Lafuente EM. et al Immune tolerance in the oral mucosa. Int J Mol Sci 2021;22:12149. 34830032 10.3390/ijms222212149 PMC8624028

[37] Murgia X , Loretz B, Hartwig O. et al The role of mucus on drug transport and its potential to affect therapeutic outcomes. Adv Drug Deliv Rev 2018;124:82–97. 29106910 10.1016/j.addr.2017.10.009

[38] Leal J , Smyth HDC, Ghosh D. Physicochemical properties of mucus and their impact on transmucosal drug delivery. Int J Pharm 2017;532:555–72. 28917986 10.1016/j.ijpharm.2017.09.018 PMC5744044

[39] Park J-Y , Chung H, DiPalma DT. et al Immune quiescence in the oral mucosa is maintained by a uniquely large population of highly activated Foxp3+ regulatory T cells. Mucosal Immunol 2018;11:1092–102. 29743613 10.1038/s41385-018-0027-2 PMC6035783

[40] Traxinger BR , Richert-Spuhler LE, Lund JM. Mucosal tissue regulatory T cells are integral in balancing immunity and tolerance at portals of antigen entry. Mucosal Immunol 2022;15:398–407. 34845322 10.1038/s41385-021-00471-x PMC8628059

[41] Hubo M , Trinschek B, Kryczanowsky F. et al Costimulatory molecules on immunogenic versus tolerogenic human dendritic cells. Front Immunol 2013;4:82. 23565116 10.3389/fimmu.2013.00082 PMC3615188

[42] Dutzan N , Abusleme L, Bridgeman H. et al On-going mechanical damage from mastication drives homeostatic Th17 cell responses at the oral barrier. Immunity 2017;46:133–47. 28087239 10.1016/j.immuni.2016.12.010 PMC5263257

[43] Chistiakov DA , Bobryshev YV, Kozarov E. et al Intestinal mucosal tolerance and impact of gut microbiota to mucosal tolerance. Front Microbiol 2014;5:781. 25628617 10.3389/fmicb.2014.00781 PMC4292724

[44] Lei C , Jiang J, Zhang Y. et al Role and function of regulatory T cell in chronic rhinosinusitis with nasal polyposis. J Immunol Res 2022;2022:1144563. 35378904 10.1155/2022/1144563 PMC8976649

[45] Golebski K , Hoepel W, van Egmond D. et al FcγRIII stimulation breaks the tolerance of human nasal epithelial cells to bacteria through cross-talk with TLR4. Mucosal Immunol 2019;12:425–33. 30664707 10.1038/s41385-018-0129-x

[46] Ryu S , Lim M, Kim J. et al Versatile roles of innate lymphoid cells at the mucosal barrier: from homeostasis to pathological inflammation. Exp Mol Med 2023;55:1845–57. 37696896 10.1038/s12276-023-01022-z PMC10545731

[47] Orimo K , Saito H, Matsumoto K. et al Innate lymphoid cells in the airways: their functions and regulators. Allergy Asthma Immunol Res 2020;12:381–98. 32141254 10.4168/aair.2020.12.3.381 PMC7061164

[48] Hsu AT , Gottschalk TA, Tsantikos E. et al The role of innate lymphoid cells in chronic respiratory diseases. Front Immunol 2021;12:733324. 34630416 10.3389/fimmu.2021.733324 PMC8492945

[49] Nagai M , Moriyama M, Ichinohe T. Oral bacteria combined with an intranasal vaccine protect from influenza A virus and SARS-CoV-2 infection. mBio 2021;12:e01598–21. 34399617 10.1128/mBio.01598-21 PMC8406166

[50] Salk HM , Simon WL, Lambert ND. et al Taxa of the nasal microbiome are associated with influenza-specific IgA response to live attenuated influenza vaccine. PLoS ONE 2016;11:e0162803. 27643883 10.1371/journal.pone.0162803 PMC5028048

[51] Hu Y , Zhang R, Li J. et al Association between gut and nasal microbiota and allergic rhinitis: a systematic review. J Asthma Allergy 2024;17:633–51. 39006241 10.2147/JAA.S472632 PMC11246088

[52] Zhao T , Li J, Fu Y. et al Influence of gut microbiota on mucosal IgA antibody response to the polio vaccine. NPJ Vaccines 2020;5:47–9. 32566258 10.1038/s41541-020-0194-5 PMC7283253

[53] Harris VC , Armah G, Fuentes S. et al Significant correlation between the infant gut microbiome and rotavirus vaccine response in rural Ghana. J Infect Dis 2017;215:34–41. 27803175 10.1093/infdis/jiw518 PMC5225256

[54] Cunningham-Oakes E , Bronowski C, Chinyama E. et al Increased bacterial taxonomic and functional diversity is associated with impaired rotavirus vaccine immunogenicity in infants from India and Malawi. BMC Microbiol 2023;23:354. 37980461 10.1186/s12866-023-03098-z PMC10656894

[55] Jonesteller CL , Burnett E, Yen C. et al Effectiveness of rotavirus vaccination: a systematic review of the first decade of global postlicensure data, 2006-2016. Clin Infect Dis Off Dis 2017;65:840–50. 10.1093/cid/cix369 28444323

[56] Lopman BA , Pitzer VE, Sarkar R. et al Understanding reduced rotavirus vaccine efficacy in low socio-economic settings. PLOS ONE 2012;7:e41720. 22879893 10.1371/journal.pone.0041720 PMC3412858

[57] Kim AH , Armah G, Dennis F. et al Enteric virome negatively affects seroconversion following oral rotavirus vaccination in a longitudinally sampled cohort of Ghanaian infants. Cell Host Microbe 2022;30:110–23.e5. 34932985 10.1016/j.chom.2021.12.002 PMC8763403

[58] Cao P , Han FY, Grøndahl L. et al Enhanced oral vaccine efficacy of polysaccharide-coated calcium phosphate nanoparticles. ACS Omega 2020;5:18185–97. 32743193 10.1021/acsomega.0c01792 PMC7392379

[59] von Halling Laier C , Gibson B, Moreno JAS. et al Microcontainers for protection of oral vaccines, *in vitro* and *in vivo* evaluation. J Control Release 2019;294:91–101. 30550938 10.1016/j.jconrel.2018.11.030

[60] Carlsen PHR , Kjeldsen RB, Pedersen GK. et al Oral vaccination using microdevices to deliver α-GalCer adjuvanted vaccine afford a mucosal immune response. J Control Release 2023;353:134–46. 36372387 10.1016/j.jconrel.2022.11.015

[61] Amin MK , Boateng J. Surface functionalization of PLGA nanoparticles for potential oral vaccine delivery targeting intestinal immune cells. Colloids Surf B Biointerfaces 2023;222:113121. 36599187 10.1016/j.colsurfb.2022.113121

[62] Davitt CJH , McNeela EA, Longet S. et al A novel adjuvanted capsule based strategy for oral vaccination against infectious diarrhoeal pathogens. J Control Release 2016;233:162–73. 27157995 10.1016/j.jconrel.2016.05.001

[63] Creighton RL , Faber KA, Tobos CI. et al Oral mucosal vaccination using integrated fiber microneedles. J Control Release 2024;367:649–60. 38295993 10.1016/j.jconrel.2024.01.062 PMC11010722

[64] Deng K , Huang Z, Jing B. et al Mucoadhesive chitosan-catechol as an efficient vaccine delivery system for intranasal immunization. Int J Biol Macromol 2024;273:133008. 38852736 10.1016/j.ijbiomac.2024.133008

[65] Lai SK , O'Hanlon DE, Harrold S. et al Rapid transport of large polymeric nanoparticles in fresh undiluted human mucus. Proc Natl Acad Sci U S A 2007;104:1482–7. 17244708 10.1073/pnas.0608611104 PMC1785284

[66] Primard C , Rochereau N, Luciani E. et al Traffic of poly(lactic acid) nanoparticulate vaccine vehicle from intestinal mucus to sub-epithelial immune competent cells. Biomaterials 2010;31:6060–8. 20471085 10.1016/j.biomaterials.2010.04.021

[67] Currie S , Kim S, Gu X. et al Mucus-penetrating PEGylated polysuccinimide-based nanocarrier for intravaginal delivery of siRNA battling sexually transmitted infections. Colloids Surf B Biointerfaces 2020;196:111287. 32768985 10.1016/j.colsurfb.2020.111287

[68] Sun S , Li E, Zhao G. et al Respiratory mucosal vaccination of peptide-poloxamine-DNA nanoparticles provides complete protection against lethal SARS-CoV-2 challenge. Biomaterials 2023;292:121907. 36436305 10.1016/j.biomaterials.2022.121907 PMC9673044

[69] Bi Q , Song X, Zhao Y. et al Mucus-penetrating nonviral gene vaccine processed in the epithelium for inducing advanced vaginal mucosal immune responses. Acta Pharm Sin B 2023;13:1287–302. 36970203 10.1016/j.apsb.2022.11.004 PMC10031263

[70] Oh S-H , Kim S-H, Jeon J-H. et al Cytoplasmic expression of a model antigen with M Cell-Targeting moiety in lactic acid bacteria and implication of the mechanism as a mucosal vaccine via oral route. Vaccine 2021;39:4072–81. 34127296 10.1016/j.vaccine.2021.06.010

[71] Mao Y , Wang X, Chen C. et al Immune-awakenin*g Saccharomyces*-inspired nanocarrier for oral target delivery to lymph and tumors. Acta Pharm Sin B 2022;12:4501–18. 36562001 10.1016/j.apsb.2022.04.018 PMC9764130

[72] Dunkin D , Berin MC, Mayer L. Allergic sensitization can be induced via multiple physiologic routes in an adjuvant-dependent manner. J Allergy Clin Immunol 2011;128:1251–8.e2. 21762973 10.1016/j.jaci.2011.06.007 PMC3197891

[73] Adel-Patient K , Bernard H, Ah-Leung S. et al Peanut- and cow’s milk-specific IgE, Th2 cells and local anaphylactic reaction are induced in Balb/c mice orally sensitized with cholera toxin. Allergy 2005;60:658–64. 15813812 10.1111/j.1398-9995.2005.00767.x

[74] Pizza M , Giuliani MM, Fontana MR. et al Mucosal vaccines: non toxic derivatives of LT and CT as mucosal adjuvants. Vaccine 2001; 19:2534–41. 11257389 10.1016/s0264-410x(00)00553-3

[75] van der Plas JL , Haijema B-J, Leenhouts K. et al Safety, reactogenicity and immunogenicity of an intranasal seasonal influenza vaccine adjuvanted with gram-positive matrix (GEM) particles (FluGEM): A randomized, double-blind, controlled, ascending dose study in healthy adults and elderly. Vaccine 2024;42:125836. 10.1016/j.vaccine.2024.03.063 38772837

[76] Pan S-C , Hsu W-T, Lee W-S. et al A double-blind, randomized controlled trial to evaluate the safety and immunogenicity of an intranasally administered trivalent inactivated influenza vaccine with the adjuvant LTh(αK): A phase II study. Vaccine 2020;38:1048–56. 31812463 10.1016/j.vaccine.2019.11.047

[77] Zhang Y , Yu X, Hou L. et al CTA1: Purified and display onto gram-positive enhancer matrix (GEM) particles as mucosal adjuvant. Protein Expr Purif 2018;141:19–24. 28866467 10.1016/j.pep.2017.08.010

[78] Sukwa N , Mubanga C, Hatyoka LM. et al Safety, tolerability, and immunogenicity of an oral inactivated ETEC vaccine (ETVAX^®^) with dmLT adjuvant in healthy adults and children in Zambia: An age descending randomised, placebo-controlled trial. Vaccine 2023;41:6884–94. 37838479 10.1016/j.vaccine.2023.09.052

[79] Hajam IA , Dar PA, Shahnawaz I. et al Bacterial flagellin—a potent immunomodulatory agent. Exp Mol Med 2017;49:e373–e373. 28860663 10.1038/emm.2017.172 PMC5628280

[80] Zhao B , Yang J, He B. et al A safe and effective mucosal RSV vaccine in mice consisting of RSV phosphoprotein and flagellin variant. Cell Rep 2021;36:109401. 10.1016/j.celrep.2021.109401 34289371

[81] Tan W , Thiruppathi J, Hong SH. et al Development of an anti-tauopathy mucosal vaccine specifically targeting pathologic conformers. Npj Vaccines 2024;9:121–16. 38879560 10.1038/s41541-024-00904-1 PMC11180213

[82] Jiang H , Zhang S, Chen Y. et al Preparation and characterization of curdlan-chitosan conjugate nanoparticles as mucosal adjuvants for intranasal influenza H1N1 subunit vaccine. Int J Biol Macromol 2024;266:131289. 38570002 10.1016/j.ijbiomac.2024.131289

[83] Hsieh H-C , Chen C-C, Chou P-H. et al Induction of neutralizing antibodies and mucosal IgA through intranasal immunization with the receptor binding domain of SARS-CoV-2 spike protein fused with the type IIb *E. coli* heat-labile enterotoxin A subunit. Antiviral Res 2023;220:105752. 37949318 10.1016/j.antiviral.2023.105752

[84] Gutjahr A , Papagno L, Nicoli F. et al The STING ligand cGAMP potentiates the efficacy of vaccine-induced CD8^+^ T cells. JCI Insight 2019;4:e125107. 10.1172/jci.insight.125107 PMC6483644 30944257

[85] Verma SK , Mahajan P, Singh NK. et al New-age vaccine adjuvants, their development, and future perspective. Front Immunol 2023;14:1043109. 36911719 10.3389/fimmu.2023.1043109 PMC9998920

[86] Varma DM , Batty CJ, Stiepel RT. et al Development of an intranasal gel for delivery of a broadly acting subunit influenza vaccine. ACS Biomater Sci Eng 2022;8:1573–82. 35353486 10.1021/acsbiomaterials.2c00015 PMC9627116

[87] Ye L , Schnepf D, Staeheli P. Interferon-λ orchestrates innate and adaptive mucosal immune responses. Nat Rev Immunol 2019;19:614–25. 31201377 10.1038/s41577-019-0182-z

[88] Ye L , Schnepf D, Becker J. et al Interferon-λ enhances adaptive mucosal immunity by boosting release of thymic stromal lymphopoietin. Nat Immunol 2019;20:593–601. 30886417 10.1038/s41590-019-0345-x

[89] Solstad AD , Denz PJ, Kenney AD. et al IFN-**λ** uniquely promotes CD8 T cell immunity against SARS-CoV-2 relative to type I IFN. JCI Insight 2024;9:e171830. 10.1172/jci.insight.171830 PMC11383353 38973611

[90] Elder E , Bangalore Revanna C, Johansson C. et al Protective immunity induced by an inhaled SARS-CoV-2 subunit vaccine. Vaccine 2023;41:4743–51. 37353452 10.1016/j.vaccine.2023.06.015 PMC10242152

[91] Kawahara M , Matsuo K, Nakasone T. et al Combined intrarectal/intradermal inoculation of recombinant *Mycobacterium bovis* bacillus Calmette–Guérin (BCG) induces enhanced immune responses against the inserted HIV-1 V3 antigen. Vaccine 2002;21:158–66. 12450689 10.1016/s0264-410x(02)00465-6

[92] Vazquez T , Torrieri-Damard L, Pitoiset F. et al Particulate antigens administrated by intranasal and intravaginal routes in a prime-boost strategy improve HIV-specific TFH generation, high-quality antibodies and long-lasting mucosal immunity. Eur J Pharm Biopharm 2023;191:124–38. 37634825 10.1016/j.ejpb.2023.08.014

[93] Paris AL , Colomb E, Verrier B. et al Sublingual vaccination and delivery systems. J Control Release Off J Control Release Soc 2021;332:553–62. 10.1016/j.jconrel.2021.03.017 33737202

[94] Shaw HA , Remmington A, McKenzie G. et al Mucosal immunization has benefits over traditional subcutaneous immunization with Group A Streptococcus antigens in a pilot study in a mouse model. Vaccines (Basel) 2023;11:1724. 38006056 10.3390/vaccines11111724 PMC10674289

[95] Laghlali G , Wiest MJ, Karadag D. et al Enhanced mucosal SARS-CoV-2 immunity after heterologous intramuscular mRNA prime/intranasal protein boost vaccination with a combination adjuvant. Mol Ther J Ther 2024;32:4448–66. 10.1016/j.ymthe.2024.10.016 PMC11638833 39489918

[96] Avanthay R , Garcia-Nicolas O, Ruggli N. et al Evaluation of a novel intramuscular prime/intranasal boost vaccination strategy against influenza in the pig model. PLOS Pathog 2024;20:e1012393. 39116029 10.1371/journal.ppat.1012393 PMC11309389

[97] Huerva V , Ascaso FJ, Grzybowski A. Ocular Inflammation. Mediators Inflamm 2015;2015:398076. 25653479 10.1155/2015/398076 PMC4310230

[98] Gery I , Caspi RR. Tolerance Induction in Relation to the Eye. Front Immunol 2018;9:2304. 30356688 10.3389/fimmu.2018.02304 PMC6189330

[99] Habot-Wilner Z , Neri P, Okada AA. et al COVID Vaccine-Associated Uveitis. Ocul Immunol Inflamm 2023;31:1198–205. 37145198 10.1080/09273948.2023.2200858

[100] Venkatesan P. Avian influenza spillover into mammals. Lancet Microbe 2023;4:e492. 37247629 10.1016/S2666-5247(23)00173-8

[101] Hikono H , Mase M, Matsuu A. et al Intraocular vaccination with an inactivated highly pathogenic avian influenza virus induces protective antibody responses in chickens. Vet Immunol Immunopathol 2013;151:83–9. 23159237 10.1016/j.vetimm.2012.10.005

[102] Yaguchi K , Ohgitani T, Noro T. et al Vaccination of chickens with liposomal inactivated avian pathogenic Escherichia coli (APEC) vaccine by eye drop or coarse spray administration. Avian Dis 2009;53:245–9. 19630231 10.1637/8475-092908-Reg.1

[103] Parez N , Fourgeux C, Mohamed A. et al Rectal immunization with rotavirus virus-like particles induces systemic and mucosal humoral immune responses and protects mice against rotavirus infection. J Virol 2006;80:1752–61. 16439532 10.1128/JVI.80.4.1752-1761.2006 PMC1367137

[104] Abolhassani M , Lagranderie M, Chavarot P. et al Mycobacterium bovis BCG induces similar immune responses and protection by rectal and parenteral immunization routes. Infect Immun 2000;68:5657–62. 10992467 10.1128/iai.68.10.5657-5662.2000 PMC101519

[105] Mayr UB , Kudela P, Atrasheuskaya A. et al Rectal single dose immunization of mice with Escherichia coli O157: H7 bacterial ghosts induces efficient humoral and cellular immune responses and protects against the lethal heterologous challenge. Microb Biotechnol 2012;5:283–94. 22103353 10.1111/j.1751-7915.2011.00316.x PMC3815788

[106] Kim-Schulze S , Kim HS, Wainstein A. et al Intrarectal vaccination with recombinant vaccinia virus expressing carcinoembronic antigen induces mucosal and systemic immunity and prevents progression of colorectal cancer. J Immunol Baltim Immunol 2008;181:8112–9. 10.4049/jimmunol.181.11.8112 19018004

[107] Varotto-Boccazzi I , Epis S, Cattaneo GM. et al Rectal administration of Leishmania cells elicits a specific, Th1-associated IgG2a response in mice: new perspectives for mucosal vaccination against Leishmaniasis, after the repurposing of a study on an anti-viral vaccine candidate. Trop Med Infect Dis 2023;8:406. 37624344 10.3390/tropicalmed8080406 PMC10458511

